# SREBP1/FASN/cholesterol axis facilitates radioresistance in colorectal cancer

**DOI:** 10.1002/2211-5463.13137

**Published:** 2021-05-01

**Authors:** Yuxiao Jin, Zhiyuan Chen, Jiali Dong, Bin Wang, Saijun Fan, Xiaodong Yang, Ming Cui

**Affiliations:** ^1^ Department of General Surgery The Second Affiliated Hospital of Soochow University Suzhou China; ^2^ Tianjin Key Laboratory of Radiation Medicine and Molecular Nuclear Medicine Institute of Radiation Medicine Chinese Academy of Medical Sciences and Peking Union Medical College Tianjin China

**Keywords:** cholesterol, colorectal cancer, radioresistance, SREBP1/FASN signaling

## Abstract

Acquired and intrinsic radioresistance remains a major challenge during the treatment of patients with colorectal cancer (CRC). Aberrant cholesterol metabolism precipitates the development of multiple cancers. Here, we report that exogenous or endogenous cholesterol enhances the radioresistance of CRC cells. The addition of cholesterol protects CRC cells against irradiation both *in vitro* and *in vivo*. Sterol response element‐binding protein 1/fatty acid synthase (SREBP1/FASN) signaling is rapidly increased in response to radiation stimuli, resulting in cholesterol accumulation, cell proliferation and inhibition of apoptosis. Blocking the SREBP1/FASN pathway impedes cholesterol synthesis and accelerates radiation‐induced CRC cell death. Our findings provide novel insights into the role of the SREBP1/FASN/cholesterol axis in radiotherapy and suggest that it may be a potential target for CRC treatment. Clinically, our results suggest that CRC patients undergoing radiotherapy may benefit from a lowered cholesterol intake.

## Graphical abstract

The findings of the present study suggest that exogenous addition of cholesterol promotes radioresistance of colorectal cancer (CRC) cells. Irradiation activates sterol response element‐binding protein 1/fatty acid synthase (SREBP1/FASN) signaling and leads to a prolonged increase of intracellular cholesterol in CRC cells. In turn, elevated cholesterol levels promote radioresistance. Therefore, therapies that target SREBP1 and FASN coupled with a low‐cholesterol diet may have potential for improving the response of patients with CRC to radiotherapy.

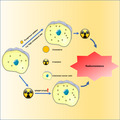

**Abbreviations**
CCK‐8: Cell Counting Kit‐8
CRC: colorectal cancer
EdU: 5‐ethynyl‐2′‐deoxyuridine
FASN: fatty acid synthase
qRT‐PCR: quantitative real‐time PCR
siRNA: small interfering RNA
SREBP1: sterol response element‐binding protein 1

Cancer’s intertwined financial burden and threat to community health represents a serious public problem worldwide. Approximately 10% of new cancer cases and cancer deaths comprose colorectal cancer (CRC), which is ranked third with respect to cancer morbidity and mortality in both sexes [1, 2]. Radiotherapy as a milestone of cancer therapy is used alone or in conjunction with other remedies in both benign and malignant neoplasms for curative or palliative purposes [3]. Radiotherapy in combination with surgery is regarded as a revolutionary treatment for CRC, especially for rectal cancer and metastatic CRC [4]. However, as a result of the radioresistant gene‐expression signature of CRC, many patients demonstrate resistance to radiotherapy, which halts the treatment and degrades the life quality of patients [5]. Thus, untangling the underlying mechanisms of radiation resistance in colorectal cancer and developing a safe and effective radiosensitizer remain unmet medical needs.

Lipid metabolism is closely correlated to the physiologic and pathologic status of individuals. In tumors, the aberrant lipid metabolism profile of cancer cell bolsters the rapid proliferation [6]. As a member of the lipids, cholesterol propels tumorigenesis, covering liver cancer, prostate cancer, breast cancer and CRC [7, 8, 9, 10]. In addition, high‐cholesterol intake not only inhibits apoptosis of cancer cells, but also precipitates metastasis [11, 12]. The sterol response element‐binding protein (SREBP) family governs the synthesis and import of cholesterol [13], and the dysregulation of SREBPs is observed in various cancers [14]. To date, the role of the SREBP/cholesterol axis in radiotherapy for CRC remains poorly understood.

Radioresistance is classified as acquired and intrinsic radioresistance [15]. In CRC, more than 70% of patients exhibit a poor response to radiotherapy, for which the factors are polymodal, finally resulting in intrinsic and acquired resistance [16]. Colorectal cancer carries a radioresistant gene‐expression signature sustaining the intrinsic radioresistance. In the present study, we aimed to investigate whether cholesterol facilitated the acquired radioresistance of CRC. Our observations indicated that exogenous cholesterol protected CRC cells against irradiation both *in vitro* and *in vivo*. SREBP1/fatty acid synthase signaling responded to radiation exposure rapidly and actuated cholesterol synthesis, resulting in the acquired radioresistance of CRC cells. Importantly, TVB‐2640, an inhibitor of FASN, might be employed as a safe and effective radiosensitizer for CRC treatment in pre‐clinical settings.

## Materials and methods

### Cell culture

Human colorectal cancer cell lines HT‐29 and HCT‐8 were purchased from the American Type Culture Collection (Manassas, VA, USA) and certified to be mycoplasma‐free. The HT‐29 and HCT‐8 cell lines, respectively, were cultured in RPMI‐1640 (Gibco, Thermo Fisher, Waltham, MA, USA) and McCoy's 5A (Gibco) supplemented with 10% FBS and 1% penicillin‐streptomycin at 5% CO_2_ and 37 °C. The experiments were performed during the period of exponential cell growth.

### Radiation study

An Exposure Instrument Gammacell‐40 ^137^Cs‐irradiator (Atomic Energy of Canadian Inc., Mississauga, ON, Canada) at a dose rate of 0.88 Gy min^–1^ was used for all of the experiments.

### Cell Counting Kit‐8 assay

The 96‐well plates were inoculated with 3000 cells per well and then exposed to 6 Gy irradiation, followed by treatment with cholesterol (2 μm) or TVB‐2640 (7.5 µg·mL^−1^) within 1 h. After 0, 1, 2, 3 days, 5 μL of Cell Counting Kit‐8 (CCK‐8) solution (Dojindo Laboratories, Kumamoto, Japan) was added into each well, followed by incubation in a cell incubator for 2 h. The absorbance was measured at a wavelength of 450 nm using an EnSpire Multimode Plate Reader (PerkinElmer, Waltham, MA, USA).

### Colony formation assay

The cells were inoculated in six‐well plates for 1000 cells per well and then exposed to 6 Gy irradiation, followed by treatment with cholesterol (or TVB‐2640) at a concentration of 2 μm (or 7.5 μg·mL^−1^). The cells were cultured in incubator for 2 weeks, with a change of fresh medium every 3 days. Colonies were washed twice with PBS, fixed with methanol for 30 min at 4 °C and then stained with Giemsa.

### 5‐ethynyl‐2′‐deoxyuridine (EdU) incorporation assay

Cells were inoculated in 24‐well plates for 12 000 cells per well and then exposed to 6 Gy irradiation, followed by treatment with cholesterol at a concentration of 2 μm. The assay was performed using the Cell‐Light TM EdU imaging detection kit (RiboBio, Guangzhou, China) in accordance with the manufacturer’s instructions.

### RNA extraction and quantitative real‐time PCR (qRT‐PCR)

Total RNA was isolated from the cells using Trizol (Invitrogen, Carlsbad, CA, USA) in accordance with the manufacturer’s instructions. Reverse transcription was performed using poly (A)‐tailed total RNA and primer with ImPro‐II Reverse Transcriptase (Promega, Madison, WI, USA) in accordance with the manufacturer’s instructions. The qRT‐PCR was performed using SYBR‐Green Mix and detected by the ABI PRISM 7500 Sequence Detection System (Applied Biosystems, Foster City, CA, USA) in accordance with the manufacturer’s instructions. Primers are listed in Table S1.

### Western blotting

The cells were lysed in RIPA buffer at 4 °C for 30 min, followed by centrifugal collection for western blotting. Proteins were separated by 8% (or 10%) SDS/PAGE and transferred to poly(vinylidene difluoride) membranes. The membranes were blocked with 5% milk at 37 °C for 1 h and incubated with primary antibodies at 4 °C overnight, and then incubated with secondary antibodies at 37 °C for 1 h. All antibodies were diluted with 5% milk: GAPDH (dilution 1 : 5000; ProteinTech Group, Chicago, IL, USA), β‐actin (dilution 1 : 5000; ProteinTech Group), SREBP1 (dilution 1 : 1000; Abcam, Cambridge, UK), FASN (dilution 1 : 1000; Santa Cruz, CA, USA) and caspase‐6 (dilution 1 : 1000; Abcam).

### Total cholesterol and triglyceride assay

The levels of cholesterol and triglyceride in cellular or tumor tissues (from nude mice) were assessed using total cholesterol assay kit E1015 (Applygen Technologies Inc., Beijing, China) and triglyceride assay kit E1013 (Applygen Technologies Inc.). All experiments were performed in accordance with the manufacturer’s instructions. In detail, 0.1 mL of lysis buffer was added to 1 × 10^6^ cells, and then 10 µL lysis buffer was added to each 1 mg of tissue.

### Cell transfection

The cells were inoculated in six‐well plates and the transfection experiments were performed when the cells reached 30–50% confluence. All transfection experiments used RFect small interfering RNA (siRNA)/miRNA Transfection Reagent (BIO‐TRAN, Changzhou, China) in accordance with the manufacturer’s instructions. Transfection efficiency was detected by qRT‐PCR and western blotting 24 h post‐transfection. The siRNA targeting SREBP1 and control siRNAs were purchased from RioBio. The sequence was: 5′‐ AAGACAGCAGAUUUAUUCAGCUUUG‐3′.

### Flow cytometry analysis

For the apoptosis assay, cells were inoculated in six‐well plates and exposed to irradiation 24 h post‐transfection, followed by the addition of siRNA (5 nm) or TVB‐2640 and subsequent culture for 24 h. Next, the cells were washed twice with ice‐cold PBS, resuspended with binding buffer (BD Bioscience, Franklin Lakes, NJ, USA) and co‐stained with AnnexinV–FITC (BD Bioscience) and prodidium iodide staining solution (BD Bioscience). Cells were cultured in darkness at 25 °C for 15 min, then analyzed by flow cytometer.

### *In vivo* experiments

For this, 4–6‐week‐old male BALB/c nu/nu nude mice were purchased from Vital River (Beijing, China) and housed in a specific pathogen‐free animal facility. All animal studies were approved by the Animal Care and Ethics Committee of the Institute of Radiation Medicine of Peking Union Medical College. Then, 4 × 10^6^ HCT‐8 cells in 0.15 mL of sterile PBS were injected subcutaneously into the armpit of mice. For the mice model regarding the impact of cholesterol on radiotherapy, BALB/c nu/nu nude mice were randomly divided into three groups (each group, *n *= 5) [Group‐I (vehicle control): saline as control; Group‐II: saline + local irradiation; Group‐III: saline + 3% cholesterol + local irradiation). For the impact of TVB‐2640 on radiotherapy, BALB/c nu/nu nude mice were randomly divided into three groups (each group, *n* = 5) [Group‐I (vehicle): saline contained 0.5% DMSO as control; Group‐II: saline + 0.5% DMSO + local irradiation; Group‐III: saline + 0.5% DMSO and 7.5 μg of TVB‐2640 + local irradiation]. Mice were treated (200 μL) via the oral route after each irradiation. Fractionated radiation treatment (3 Gy per day) was given until a cumulative dose of 12 Gy was achieved. Tumor size was measured periodically. Tumor volume was monitored by measuring the length (*L*) and width (*W*) of the tumors and was calculated using: (*L* × *W* ^2^) × 0.5.

### Immunohistochemistry staining

Following death of the mice, tumors were fixed in 4% neutral buffered formalin overnight at room temperature and then embedded in paraffin. These tissues were sectioned at 5 μm thickness, dewaxed, blocked with goat serum albumin for 1 h at 37 °C and incubated with primary antibodies overnight at 4 °C. On the second day, these tissues were incubated with secondary antibodies for 1 h at 37 °C, stained with diaminobenzidine and, finally, counterstained with hematoxylin. The reagents employed were: Ki‐67 (dilution 1 : 200; ProteinTech Group), p27 (dilution 1 : 100; Santa Cruz, CA, USA), caspase‐6 (dilution 1:100; Abcam) and horseradish peroxidase‐conjugated secondary antibodies (ZSGB‐BIO, Beijing, China). The area of positive staining was analyzed using image‐pro plus, version 6.0 (Media Cybernetics, Inc., Rockville, MD, USA).

### Statistical analysis

Each experiment was performed at least three times. Student's *t*‐test was used to analyze the data, which is reported as the mean ± SD. *P* < 0.05 was considered statistically significant.

## Results

### *Cholesterol protects CRC cells against radiation challenge both* in vitro *and* in vivo

**Fig. 1:**
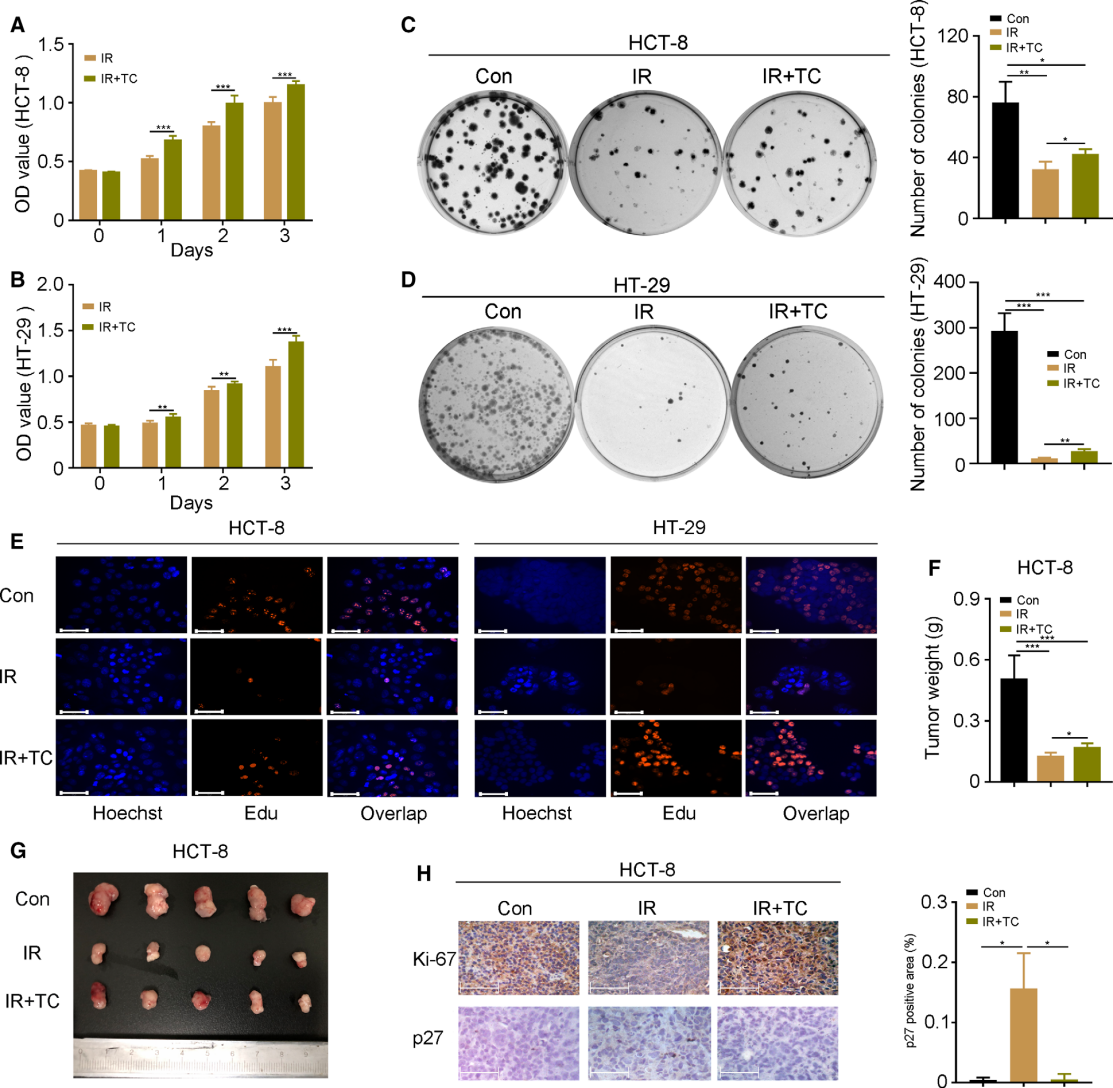
Cholesterol protects CRC cells against radiation challenge both *in vitro* and *in vivo*. (A–E) The effects of cholesterol (2 µm) on the proliferation of HCT‐8 and HT‐29 cells were assessed by CCK‐8, colony formation and EdU incorporation assays following 6 Gy γ‐ray radiation challenge. Scale bars = 125 μm. An EdU incorporation assay was performed at 24 h after 6 Gy γ‐ray irradiation. (F, G) Weight and a photograph of HCT‐8 tumors from nude mice after total 12 Gy γ‐ray irradiation. (H) The expression of Ki‐67 and p27 was detected by immunohistochemistry staining in HCT‐8 tumors from nude mice. Scale bars = 125 μm. The p27 positive area was analyzed using image‐pro plus. Data are shown as the mean ± SD. Statistical significance: ^*^ *P* < 0.05; **P < 0.01; ***P < 0.001, Student’s *t‐*test.

To address the role of cholesterol in radiotherapy for CRC, exogenous cholesterol was added into cell culture media after employing 6 Gy γ‐ray irradiation. CCK‐8, colony formation and EdU incorporation assays showed that cholesterol addition facilitated the proliferation of irradiated HCT‐8 and HT‐29 cells (Fig. 1A–E). Then, BALB/c athymic nude mice were injected with HCT‐8 cells subcutaneously and exposed to five fractions of irradiation (12 Gy in toyal) with or without cholesterol administration via the oral route. As expected, oral gavage of cholesterol increased the tumor weight (Fig. 1F) and volume (Fig. 1G), suggesting that cholesterol enhances CRC cell survival and proliferation following radiation exposure. In addition, immunohistochemistry staining further validated that the expression of Ki‐67 (a marker of proliferation) was up‐regulated and p27 (a tumor suppressor gene) was down‐regulated following cholesterol treatment (Fig. 1H). Taken together, our findings indicate that exogenous cholesterol enhances the acquired radioresistance of CRC cells.

### Radiation exposure precipitates cholesterol synthesis through SREBP1/FASN signaling in CRC cells

**Fig. 2:**
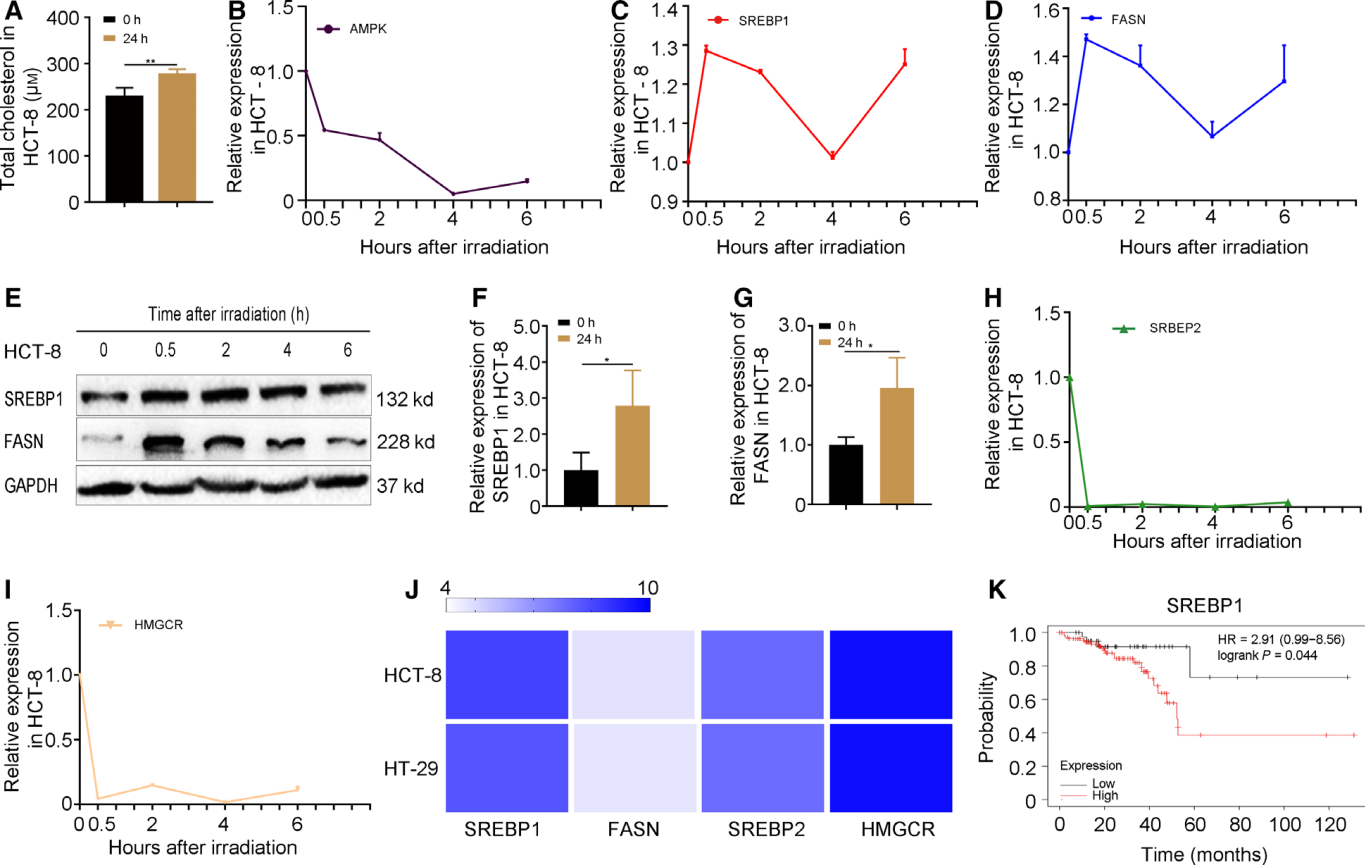
Radiation exposure precipitates cholesterol synthesis through SREBP1/FASN signaling in CRC cells. (A) The level of cholesterol in HCT‐8 cells at 24 h after 6 Gy γ‐ray irradiation. (B) The dynamic expression of AMPK was examined by qRT‐PCR at 0, 0.5, 2, 4 and 6 h after 6 Gy γ‐ray irradiation in HCT‐8 cells. (C–E) The dynamic expression of SREBP1 and FASN was examined by qRT‐PCR at 0, 0.5, 2, 4 and 6 h after 6 Gy γ‐ray irradiation in HCT‐8 cells. (F, G) The expression of SREBP1 and FASN was examined by qRT‐PCR at 24 h after 6 Gy γ‐ray irradiation in HCT‐8 cells. (H, I) The dynamic expression of SREBP2 and HMGCR was examined by western blotting at 0, 0.5, 2, 4 and 6 h after 6 Gy γ‐ray irradiation in HCT‐8 cells. (J) The basal expression of SREBP1/FASN and SREBP2/HMGCR in HCT‐8 and HT‐29 cell lines was analyzed by ccle (https://portals.broadinstitute.org/ccle). Color depth represents the intensity of expression. (K) Kaplan–Meier analysis of the overall survival rate of rectum adenocarcinoma patients (http://kmplot.com/analysis). Data are shown as the mean ± SD. GAPDH was used as a loading control. Statistical significance: **P < 0.01; ***P < 0.001, Student’s *t*‐test.

Next, we assessed the intracellular cholesterol level in HCT‐8 cells following irradiation. Intriguingly, radiation challenge elevated the cholesterol level at 24 h after irradiation (Fig. 2A). AMPK is a negative regulator for the SREBP family and inhibits cholesterol synthesis. Thus, we examined the expression of AMPK post‐irradiation. The qRT‐PCR assay revealed that radiation stimuli down‐regulated AMPK within 6 h (Fig. 2B). Then, we examined the expression pattern of SREBP1/FASN and SREBP2/HMGCR signaling in HCT‐8 cells. Notably, SREBP1 and its downstream gene FASN exhibit a possible upward trend by radiation stimuli within 6 h (Fig. 2C–E). We also found that the expression of SREBP1/FASN was maintained at a high level at 24 h post‐irradiation (Fig 2F and G and Fig. S1A). However, SREBP2/HMGCR signaling exhibited an absolute downward trend (Fig. 2H and I), implying that SREBP1/FASN might contain rapid response genes for irradiation. We repeated the experiments in HT‐29 cells and obtained similar results (Fig. S1B–H). We also assessed the triglyceride levels in HCT‐8 and HT‐29 cells. The results obtained showed that irradiation reduced triglyceride in the cell lines (Fig. S1I and J), suggesting that SREBP1 deactivates triglyceride synthesis in radiation settings. Subsequently, the basal expression of SREBP1/FASN and SREBP2/HMGCR in HCT‐8 and HT‐29 cells was analyzed by ccle (https://portals.broadinstitute.org/ccle). Although SREBP2/HMGCR and SREBP1 showed high expression, FASN exhibited an overtly low level in the two cell lines, indicating that SREBP1/FASN signaling is non‐activated in CRC cells without stimulation (Fig. 2J). Finally, we analyzed the overall survival rate of rectum adenocarcinoma patients based on the expression of SREBP1 (http://kmplot.com/analysis). Intriguingly, rectum adenocarcinoma patients with high level of SREBP1 demonstrated a lower overall survival rate (Fig. 2K). Taken together, our observations demonstrate that radiation exposure activates SREBP1/FASN signaling, elevating intracellular cholesterol in CRC cells.

### Silencing SREBP1 sensitizes CRC cells to irradiation and accelerates apoptosis

**Fig. 3:**
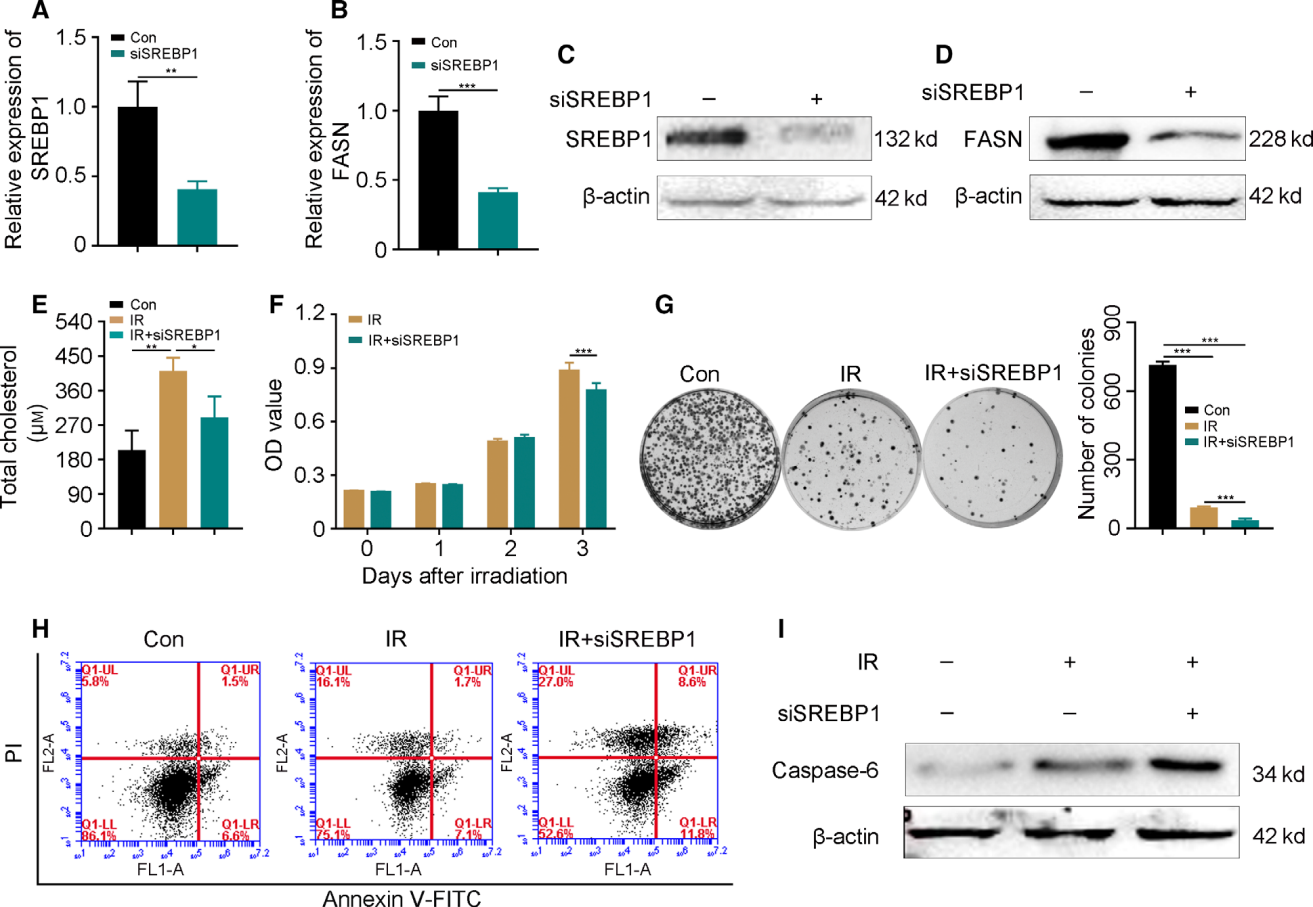
Silencing SREBP1 sensitizes CRC cells to irradiation and accelerates apoptosis. (A–D) The expression of SREBP1 and FASN after the interference of 5 nm siRNA targeting SREBP1 was examined by qRT‐PCR and western blotting in HCT‐8 cells. (E) The level of cholesterol in cells with siSREBP1 interference 24 h post‐irradiation. (F, G) The proliferation of HCT‐8 cells with or without siSREBP1 interference was assessed by (F) CCK‐8 and (G) colony formation assays following radiation challenge. (H) Flow cytometric analysis showed the apoptosis of HCT‐8 cells with or without siSREBP1 treatment at 24 h after 6 Gy γ‐ray irradiation. (I) The expression of caspase‐6 was examined by western blotting in HCT‐8 cells with or without siSREBP1 treatment at 24 h after 6 Gy γ‐ray irradiation. Data are shown as the mean ± SD. β‐actin was used as a loading control. Statistical significance: *P < 0.05; **P < 0.01; ***P < 0.001, Student’s *t*‐test.

To determine whether the radiation‐activated SREBP1/FASN/cholesterol axis contributes to the acquired radioresistance of CRC cells, we transfected siRNA targeting SREBP1 (termed as siSREBP1) into HCT‐8 cells. As shown in Fig. 3A–D, siRNA transfection indeed decreased the expression of SREBP1 and its downstream gene FASN. As expected, the cholesterol level was also reduced, combined with the deletion of SREBP1 in the irradiated cells (Fig. 3E), which supported the results indicating that radiation‐elevated SREBP1/FASN signaling precipitates cholesterol synthesis in CRC cells. CCK‐8 and colony formation assays revealed that deletion of SREBP1 hindered the proliferation of HCT‐8 cells following irradiation (Fig. 3F and G). Flow cytometric analysis further showed that radiation challenge increased the number of apoptotic HCT‐8 cells slightly (early apoptosis from 6.6% to 7.1% and late apoptosis from 1.5% to 1.7%); however, deletion of SREBP1 accelerated apoptosis (early apoptosis from 7.1% to 11.8% and late apoptosis from 1.7% to 8.6%, Fig. 3H). Western blotting confirmed that the expression of xaspase‐6 was further up‐regulated with siSREBP1 transfection following radiation stimuli (Fig. 3I), implying SREBP1/FASN signaling might be a potential target for improving the radiosensitivity of CRC cells. Taken together, our observations demonstrate that silencing of SREBP1/FASN signaling inhibits the proliferation and accelerates the apoptosis of CRC cells following irradiation.

### FASN inhibitor accelerates CRC cell death following radiation exposure

**Fig. 4:**
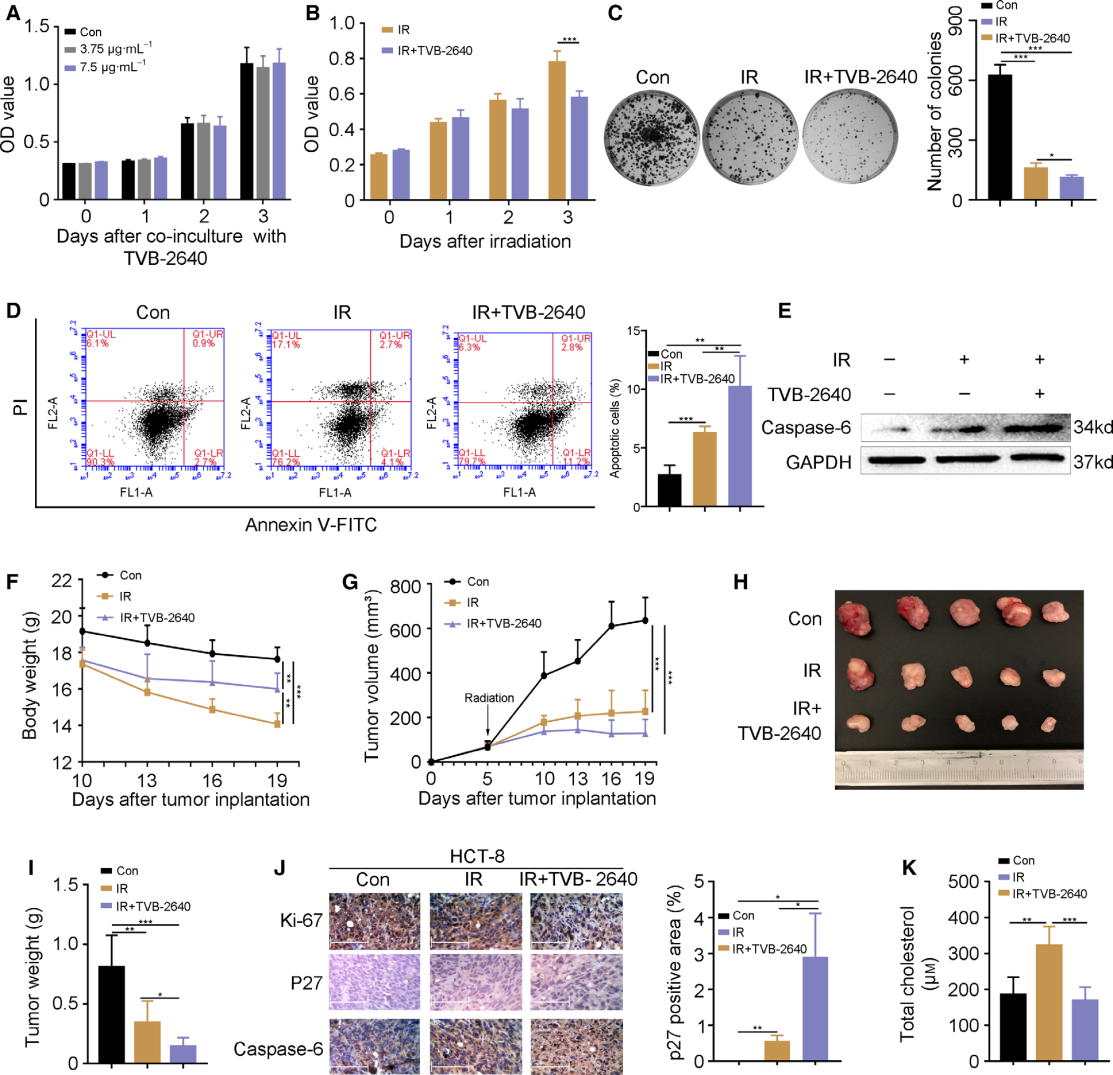
FASN inhibitor accelerates CRC cell death following radiation exposure. (A) A CCK‐8 assay showed the cell viability of HCT‐8 cells treated with TVB‐2640. (B, C) The proliferation of HCT‐8 cells with or without TVB‐2640 treatment was assessed by CCK‐8 and colony formation assays following radiation challenge. (D) Flow cytometric analysis showed the apoptosis in HCT‐8 cells treated with TVB‐2640 at 24 h after 6 Gy γ‐ray irradiation. (E) The expression of caspase‐6 was examined by western blotting in HCT‐8 cells treated with TVB‐2640 at 24 h after 6 Gy γ‐ray irradiation. (F) The body weight of nude mice. (G–I) Growth curve, photograph and weight of HCT‐8 tumors from nude mice. (J) The expression of Ki‐67, p27 and caspase‐6 was detected by immunohistochemistry staining in HCT‐8 tumors from nude mice. Scale bars = 125 μm. The p27 positive area was analyzed using image‐pro plus. (K) The level of cholesterol in HCT‐8 tumor from nude mice. Data are shown as the mean ± SD. GAPDH was used as a loading control. Statistical significance: *P < 0.05; **P < 0.01; ***P < 0.001, Student’s *t*‐test.

TVB‐2640 is a FASN inhibitor currently being used for the treatment of non‐alcoholic steatohepatitis in a clinical phase II trial. Thus, we used TVB‐2640 to further investigate whether blocking SREBP1/FASN signaling improves the efficacy of radiotherapy for CRC. TVB‐2640 showed non‐cytotoxicity to HCT‐8 cells at concentrations of 3.75 μg·mL^−1^ and 7.5 μg·mL^−1^ (Fig. 4A). Next, we used TVB‐2640 at 7.5 μg·mL^−1^ for the *in vitro* experiments. As expected, CCK‐8 and colony formation assays revealed that TVB‐2640 treatment facilitated radiation‐induced cell death of HCT‐8 cells (Fig. 4B and C). TVB‐2640 increased the number of early apoptotic HCT‐8 cells (from 4.1% to 11.2%, Fig. 4D) and up‐regulated the expression of caspase‐6 (Fig. 4E). Next, BALB/c athymic nude mice were injected with HCT‐8 cells subcutaneously and exposed to fractional local irradiation with or without TVB‐2640 administration via the oral route. Intriguingly, TVB‐2640 treatment stabilized the body weight of irradiated mice (Fig. 4F) and potentiated the tumoricidal effects of irradiation, represented by the smaller volume (Fig. 4G and H) and lighter weight (Fig. 4I) of tumors. Immunohistochemistry staining further confirmed that oral gavage of TVB‐2640 down‐regulated the expression of Ki‐67 and up‐regulated that of p27 and caspase‐6 (Fig. 4J) in tumor tissues. Finally, we assessed the level of cholesterol in the tumor tissues from nude mice. Fraction radiation exposure increased the level of cholesterol, whereas TVB‐2640 treatment restrained the elevation (Fig. 4K). Taken together, our findings demonstrate that inhibition of SREBP1/FASN signaling might represent a strategy for enhancing the efficacy of radiotherapy for CRC. TVB‐2640, an inhibitor of FASN, might be employed as a potential radiosensitizer for CRC treatment in clinical applications.

## Discussion

Obesity, poor diet and excess drinking raise the risk of CRC. These risk factors are, at least partly, associated with abnormal lipid metabolism [17]. Cholesterol, a member of lipids, is a double‐edged sword for health. On the one hand, cholesterol stabilizes the cell membrane and serves as a precursor of various substances, such as steroid hormones, bile acids and vitamin D. On the other hand, cholesterol homeostasis imbalance drives multiple diseases, covering colorectal cancer. For CRC treatment, we found that exogenous cholesterol precipitated acquired radioresistance both *in vitro* and *in vivo*. All of the evidence indicates that cholesterol degrades the efficacy of radiotherapy and emerges as a potential pitfall for CRC patients with respect to treatment. In other words, CRC patients should avoid excessive cholesterol intake during radiotherapy.

Clinically, alimentary canal neoplasm exhibits overt acquired and intrinsic resistance toward irradiation, limiting the application of radiotherapy [16, 18]. However, the underlying mechanism remains poorly understood. Recent research reports that cholesterol synthesis is implemented at 1 week post‐irradiation in lung cancer [19]. In parallel, our findings showed that the endogenous cholesterol was elevated in CRC cells after 24 h of radiation exposure. In light of these results, the endogenous cholesterol accumulation in the early post‐irradiation may account for the higher acquired radioresistance of CRC. The consensus is that traditional, SREBP2/HMGCR signaling is the major modulator for cholesterol synthesis and SREBP1/FASN signaling governs fatty acid synthesis in various cancers [13]. Recently, increasing studies report that SREBP1/FASN axis also participates in the synthesis of cholesterol under specific conditions [20, 21]. Thus, we assessed the expression of SREBP2/HMGCR and SREBP1/FASN signaling pathways in the present study. Intriguingly, SREBP1/FASN signaling, and not the SREBP2/HMGCR axis, was activated in CRC cells within 6 h after radiation stimuli, indicating that the SREBP1/FASN pathway is a rapid and direct responder for irradiation. ccle analysis showed high basal expression of SREBP1 and SREBP2 in HCT‐8 and HT‐29 cell lines; however, their downstream genes, FASN and HMGCR, represented a different basal level. The SREBP family, as a transcription factor, exerts its function in a nuclear localization‐dependent manner after activation [22], which is partly reflected in the up‐regulation of its downstream gene at the transcriptional level. Thus, the results from ccle imply that SREBP1 might be located in cytoplasm in HCT‐8 and HT‐29 cell lines, resulting in the inactivation of SREBP1/FASN signaling without stimuli. In addition, restraining SREBP1/FASN signaling decreased cholesterol synthesis, which was accompanied by inhibition of proliferation and promotion of apoptosis in CRC cells after radiation exposure both *in vitro* and *in vivo*. In this regard, the activation of SREBP1/FASN signaling by irradiation elevated cholesterol synthesis for a long time, facilitating CRC cell proliferation and apoptosis inhibition. Such evidence supports the idea that, under radiation stimuli, the SREBP1/FASN/cholesterol axis is activated, eliciting an acquired radioresistance of CRC cells. Clinically, increasing the cumulative dose in an attempt to improve the curative effects of radiotherapy might aggravate the adverse side effects and even enhance the invasion of cancer [23]. SREBP1 and FASN have been reported to exacerbate the invasion and metastasis of CRC [24, 25]. In light of our findings, the SREBP1/FASN pathway is a key target for CRC treatment. Blockade of this axis might improve the efficacy and prognosis of radiotherapy for CRC patients. Data from phase Ⅰ clinical trial corroborate that TVB‐2640, a FASN inhibitor, restrains the uptrend of hepatic *de novo* lipogenesis and thus emerges as a novel therapeutic avenue for non‐alcoholic fatty liver disease. Moreover, studies show that a combination of TVB‐2640 and paclitaxel in non‐small cell lung carcinoma and breast cancer patients improves the prognosis. TVB‐2640 is the only FASN inhibitor to have been moved into the clinic to date [26]. Thus, we assessed the synergistic effects of TVB‐2640 in radiotherapy for CRC. As expected, TVB‐2640 elevated the radiosensitivity of CRC cells, as judged by restraining cell proliferation and accelerating apoptosis following radiation exposure. Importantly, oral gavage of TVB‐2640 reduced the weight loss of irradiated mice effectively, indicating that TVB‐2640 might be employed as a safe and effective radiosensitizer for CRC treatment, athough further studies are warranted.

## Conflict of interest

The authors declare that they have no conflict of interest.

## Author contributions

YJ, ZC, MC and XY conceived or supervised the study. YJ, ZC and MC designed experiments. YJ, ZC, JD and BW performed experiments. SF, MC and XY provided new tools and reagents. YJ and ZC analysed data. YJ wrote the manuscript. ZC, MC, JD and BW made manuscript revisions.

## Supporting information


**Table S1.** Primer sequences used for qRT‐PCR.
**Fig. S1.**Radiation exposure precipitates cholesterol synthesis through SREBP1/FASN signaling in CRC cells.

## Data Availability

Data will be made available from the corresponding author upon reasonable request.
