# Supplementary material for: SREBP1/FASN/cholesterol axis facilitates radioresistance in colorectal cancer
Source: FEBS Open Bio. 2021 May 1;11(5):1343–52. doi: 10.1002/2211-5463.13137 (PMC8091817; doi:10.1002/2211-5463.13137)
Supplement: Supplementary file 1 — Table S1. Primer sequences used for qRT‐PCR. Fig. S1.Radiation exposure precipitates cholesterol synthesis through SREBP1/FASN signaling in CRC cells. [file FEB4-11-1343-s001.docx]

**Supplementary Table 1.**

Primer s­equences used for qRT-PCR.

| **Gene** | **Primer** | **Sequence (5′-3′)** |
| --- | --- | --- |
| *GAPDH* | forward | GGAGCGAGATCCCTCCAAAAT |
|  | reverse | GGCTGTTGTCATACTTCTCATGG |
| *β-Actin* | forward | CCACGAAACTACCTTCAACTCC |
|  | reverse | GTGATCTCCTTCTGCATCCTGT |
| *AMPK* | forward | TTGAAACCTGAAAATGTCCTGCT |
|  | reverse | GGTGAGCCACAACTTGTTCTT |
| *SREBP2* | forward | TGGCTCATCTTTGACCTTTGC |
|  | reverse | GCGCCAGGAGAACATGGT |
| *HMGCR* | forward | GGACCCCTTTGCTTAGAT |
|  | reverse | CCACCAAGACCTATTGCT |
| *SREBP1* | forward | ACAGTGACTTCCCTGGCCTAT |
|  | reverse | GCATGGACGGGTACATCTTCAA |
| *FASN* | forward | GGACCTGTCTAGGTTTGATGC |
|  | reverse | TGGCTTCATAGGTGACTTCCA |

**Supplementary Figure 1**


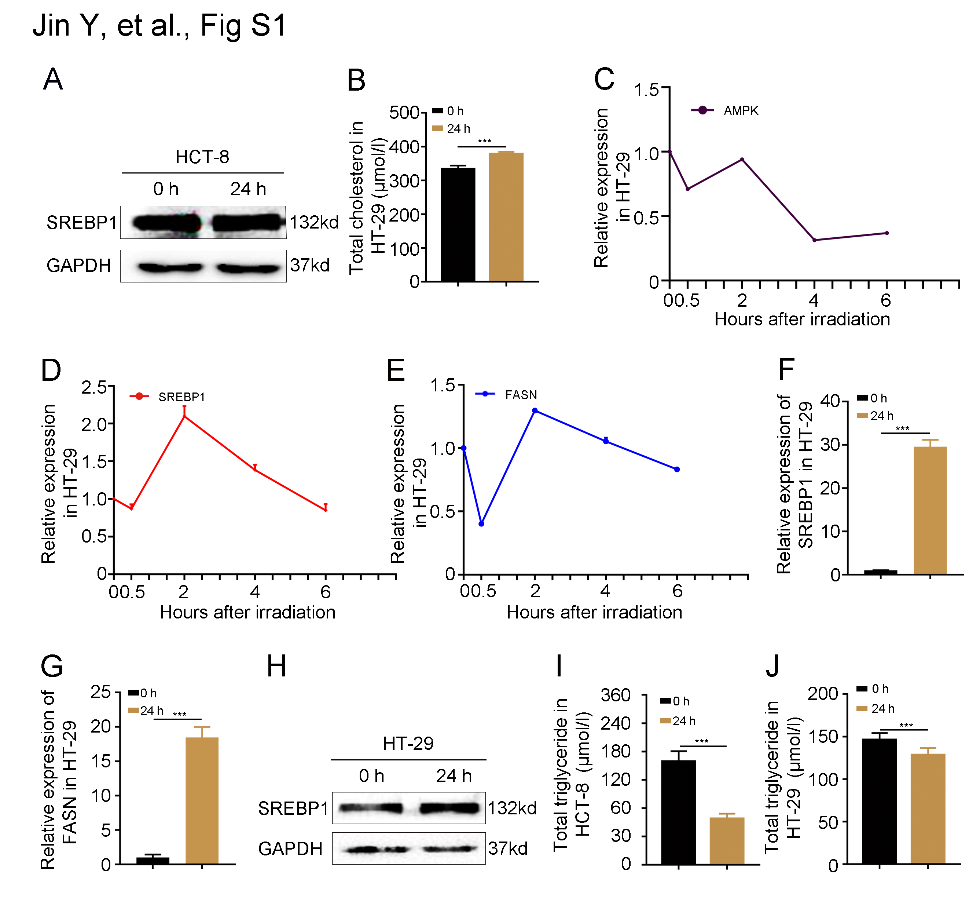


**Supplementary Figure 1. Radiation exposure precipitates cholesterol synthesis through SREBP1/FASN signaling in CRC cells.**

(A) The expression of SREBP1 was examined by Western blot at 24 h after 6 Gy γ-ray irradiation in HCT-8 cells. (B) The level of cholesterol in HT-29 cells at 24 h after 6 Gy γ-ray irradiation. (C) The dynamic expression of AMPK was examined by qRT-PCR at 0, 0.5, 2, 4 and 6 h after 6 Gy γ-ray irradiation in HT-29 cells. (D-E) The dynamic expression of SREBP1, FASN was examined by qRT-PCR at 0, 0.5, 2, 4 and 6 h after 6 Gy γ-ray irradiation in HT-29 cells. (F-G) The expression of SREBP1 and FASN was examined by qRT-PCR at 24 h after 6 Gy γ-ray irradiation in HT-29 cells. (H) The expression of SREBP1 was examined by Western blot at 24 h after 6 Gy γ-ray irradiation in HT-29 cells. (I-J) The level of triglyceride in HCT-8 and HT-29 cells at 24 h after 6 Gy γ-ray irradiation. Data showed as mean ± SD. GAPDH was as a loading control. Statistical significance: *** indicates *P* < 0.001, Student’s *t*-test.
